# In-house 3D-printed surgical guides for osseous lesions of the lower jaw: an experimental study

**DOI:** 10.1186/s40001-021-00495-w

**Published:** 2021-03-15

**Authors:** Lukas Postl, Thomas Mücke, Stefan Hunger, Oliver Bissinger, Michael Malek, Svenia Holberg, Rainer Burgkart, Stefan Krennmair

**Affiliations:** 1 Department of Oral and Maxillofacial Surgery, Kepler University Hospital GmbH, Johannes Kepler University Linz, Krankenhausstr. 9, 4021 Linz, Austria; 2 NumBioLab, Ludwig-Maximilians University of Munich, Munich, Germany; 3 Department of Oral and Maxillo-Facial Surgery, Klinikum rechts der Isar, Technische Universitaet Muenchen, Munich, Germany; 4 Department of Oral and Maxillofacial Surgery, Medizinische Universitaet Innsbruck, Innsbruck, Austria; 5 Department of Orthopaedics and Sports Orthopedics, Klinikum rechts der Isar, Technische Universitaet Muenchen, Munich, Germany

**Keywords:** 3D-printed surgical guide, Computer-assisted surgery, Computer-guided surgery, Stereolithography, Biopsy, 3D-printed bone model

## Abstract

**Background:**

The accuracy of computer-assisted biopsies at the lower jaw was compared to the accuracy of freehand biopsies.

**Methods:**

Patients with a bony lesion of the lower jaw with an indication for biopsy were prospectively enrolled. Two customized bone models per patient were produced using a 3D printer. The models of the lower jaw were fitted into a phantom head model to simulate operation room conditions. Biopsies for the study group were taken by means of surgical guides and freehand biopsies were performed for the control group.

**Results:**

The deviation of the biopsy axes from the planning was significantly less when using templates. It turned out to be 1.3 ± 0.6 mm for the biopsies with a surgical guide and 3.9 ± 1.1 mm for the freehand biopsies.

**Conclusions:**

Surgical guides allow significantly higher accuracy of biopsies. The preliminary results are promising, but clinical evaluation is necessary.

## Background

For exact diagnosis of osseous oral-maxillofacial pathologies a biopsy is required [1]. Biopsies of osseous jaw tumors can be challenging: in a series of 48 patients, it is reported that ‘Failure to obtain tumor tissue was the most common reason for incorrect diagnosis’ [2]. Besides, this it is important to preserve anatomical structures such as nerves and the periapical region of the teeth [3, 4]. Very recently, Valdec et al. published a case report about the ‘guided biopsy of osseous pathologies in the jaw bone using a 3D-printed, tooth-supported drilling template’ [4]. The case report shows that 3D-printed templates can be successfully used on patients, but since the method was only used on one patient, there are no data from a case series so far.

Therefore, this is a very newly described method and there are no data in the literature to date that indicate whether template-guided biopsies are more accurate than freehand biopsies in the region of the jaw.

However, there are several systems available that allow template-assisted placement of dental implants [5–8].

In the field of dental implantation, the literature already shows that the template method was associated with fewer errors [9, 10].

The aim of this study was to evaluate the accuracy of biopsies of the lower jaw with the help of 3D-printed surgical templates in a 3D-printed bone model of the lower jaw. The accuracy of computer-assisted biopsies was compared to the accuracy of freehand biopsies. The geometries of the 3D-printed lower jaws were identical to those of the patients, who each had a lesion at the lower jaw with an indication for biopsy. The null hypothesis (H0) is that there is no difference between the accuracy of computer-assisted biopsies and freehand biopsies of the control group.

## Methods

### Study design

The CT data of patients with a bony lesion of the lower jaw and with an indication for biopsy were used for this experimental study. The use of the CT data was approved by the local ethics committee.

### Planning and 3D printing of bone models of the lower jaw

**Fig. 1:**
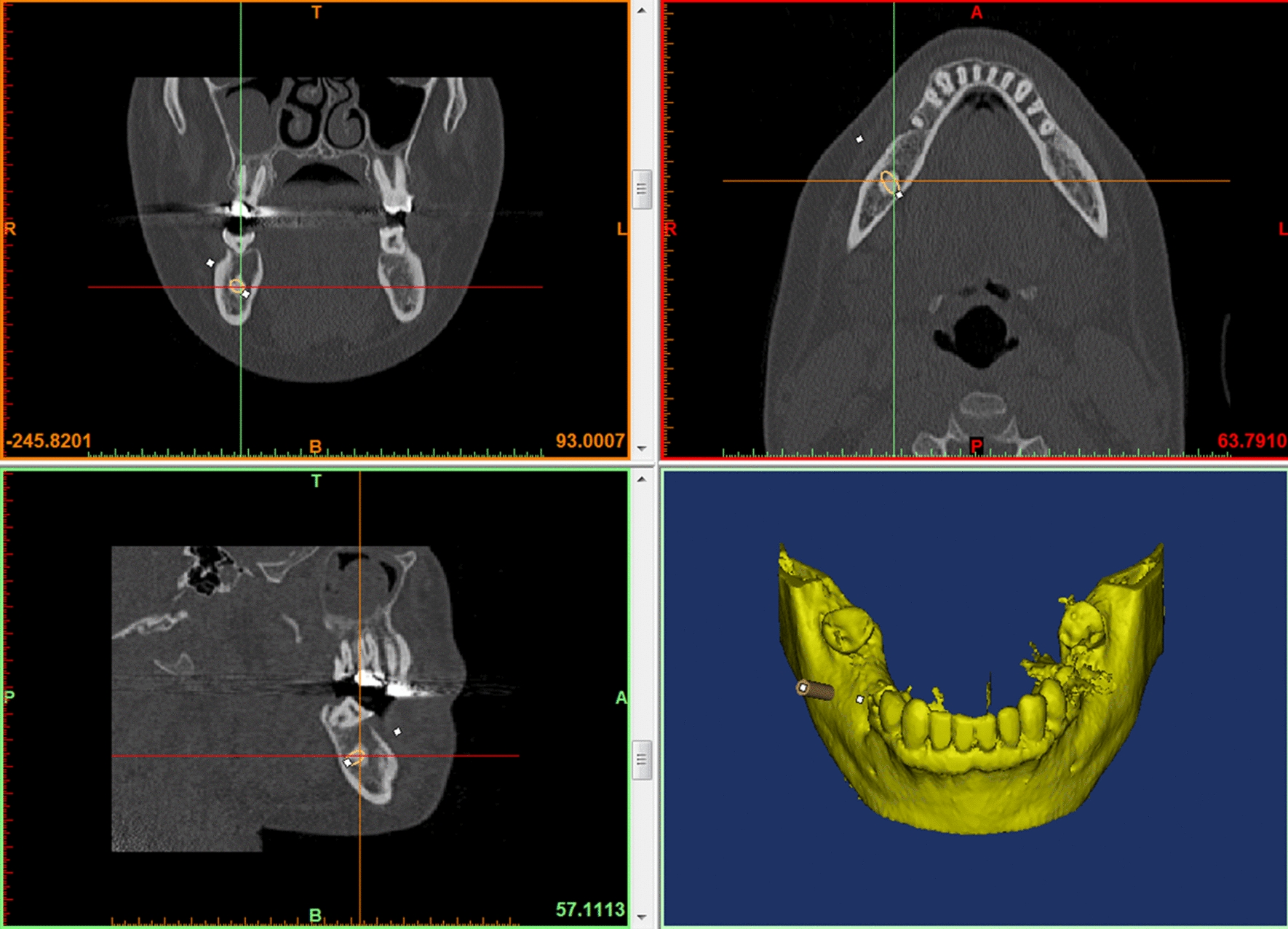
Part of a screenshot while planning of the biopsy channel according to CT data

The DICOM (Digital Imaging and Communications in Medicine) data of the CT scan was then transferred to the image segmentation and 3D model creation software Mimics Innovation Suite (Materialise, Leuven, Belgium). After segmentation, the osseous and dental information was available in STL data format. The biopsy channel was then planned considering important anatomical structures such as nerves and teeth roots (Fig. 1).

Customized bone models of the patients’ lower jaws were produced by a 3D printer (ZPrinter 650, 3D Systems, Rock Hill, South Carolina, USA) according to the previously generated CAD data (STL) of the patients’ jaw. Therefore, the bone modes of lower jaws used in this study showed identical dental and bone geometries to the patients’ lower jaws.

### CT scans before operation/experiments

**Fig. 2:**
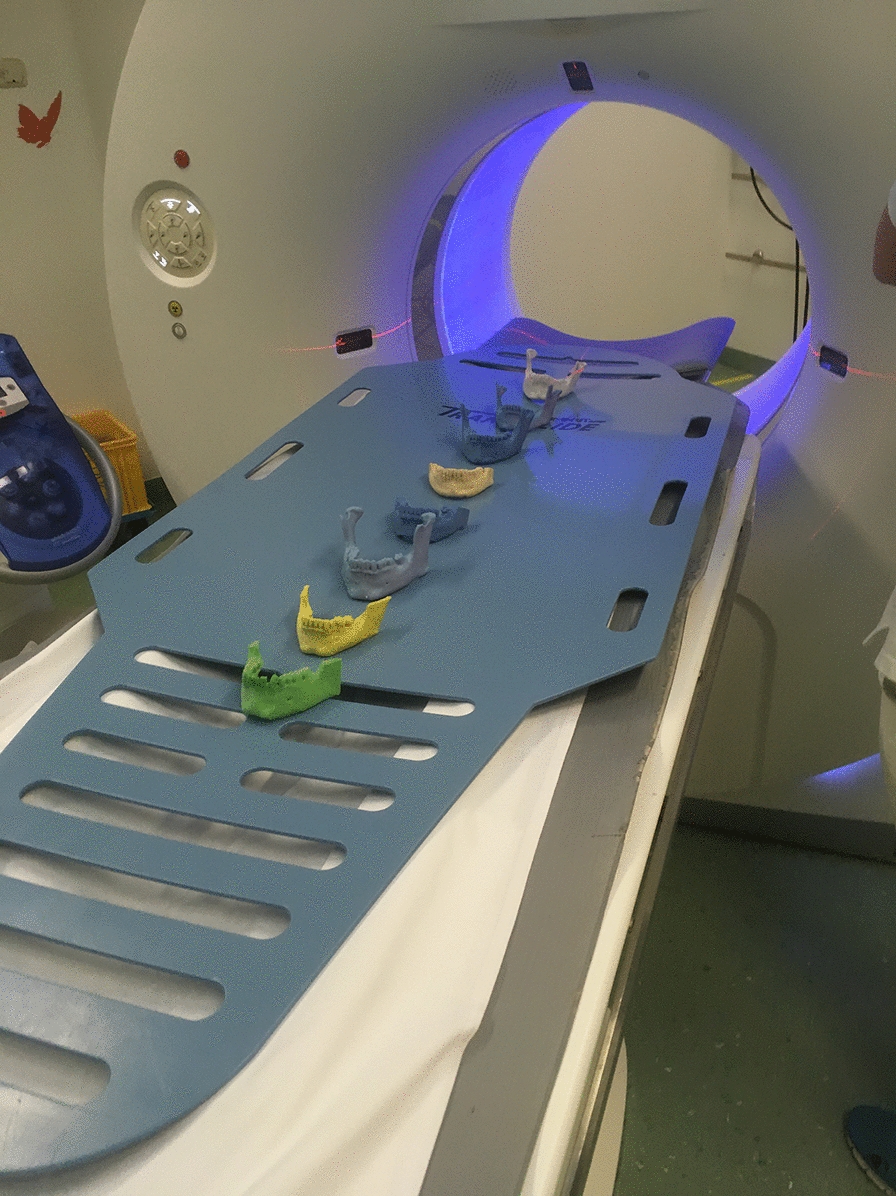
CT scans of all models of the study group

CT scans (Fig. 2) of the 3D-printed jaws (of the guided group) were performed with a Siemens Somatom Force CT scanner (120 kV, 330 mAs, collimation 64 × 0.6 mm, pitch 0.55, slice 0.75 mm).

### Design and 3D printing of surgical templates

The data of the CT scans were transferred to the image segmentation and 3D model creation tool Mimics Innovation Suite (Materialise, Leuven, Belgium). A fusion of the planning data and data of the CT scans before surgery was performed. The surgical template was then designed. The depth of the biopsy channel was determined through a limit stop: the head of the contra angle stopped at the surface of the surgical template.

**Fig. 3:**
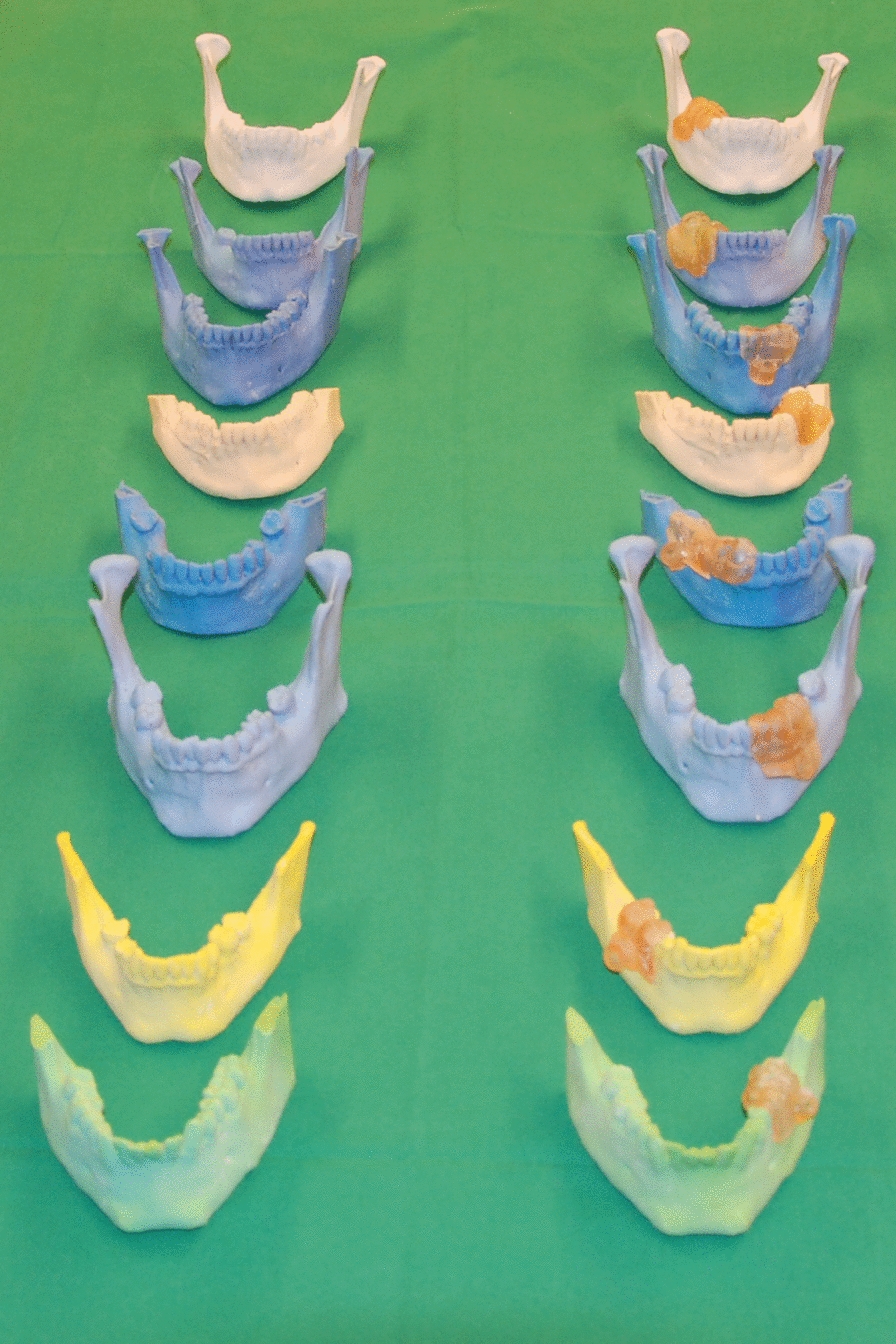
Printed models of the control group on the left side and of the study group on the right side (with surgical guides)

The STL Data of the surgical template was transferred to the software of the 3D Printer (PreForm Software, Formlabs Inc., Somerville, MA, USA) via an USB flash drive. The template was produced by the 3D printer (Form 2, Formlabs Inc., Somerville, MA, USA) using stereolithography with a class 1 biocompatible resin. The printer has an axis resolution of 0.025 mm and a build volume of 125 × 125 × 165 mm. The printer easily fits on the desktop and has a weight of 8 kg. After printing, all models and guides were available (Fig. 3).

### Biopsies with surgical guides

**Fig. 4:**
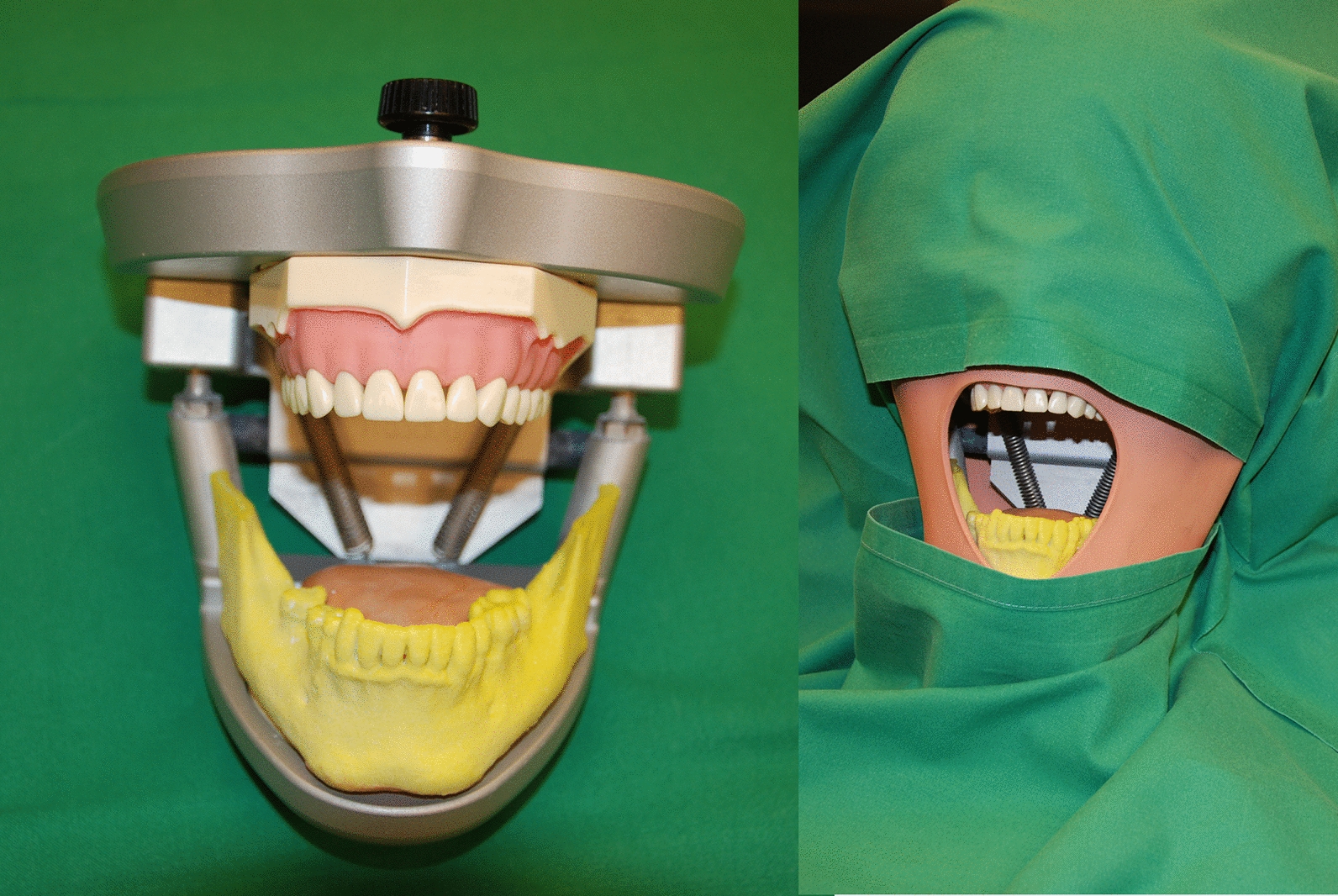
The models were mounted in a phantom head (the left side of picture shows the lower part of the phantom head). The phantom head with a soft tissue mask and upper body torso was placed on the operating table and covered in usual way

**Fig. 5:**
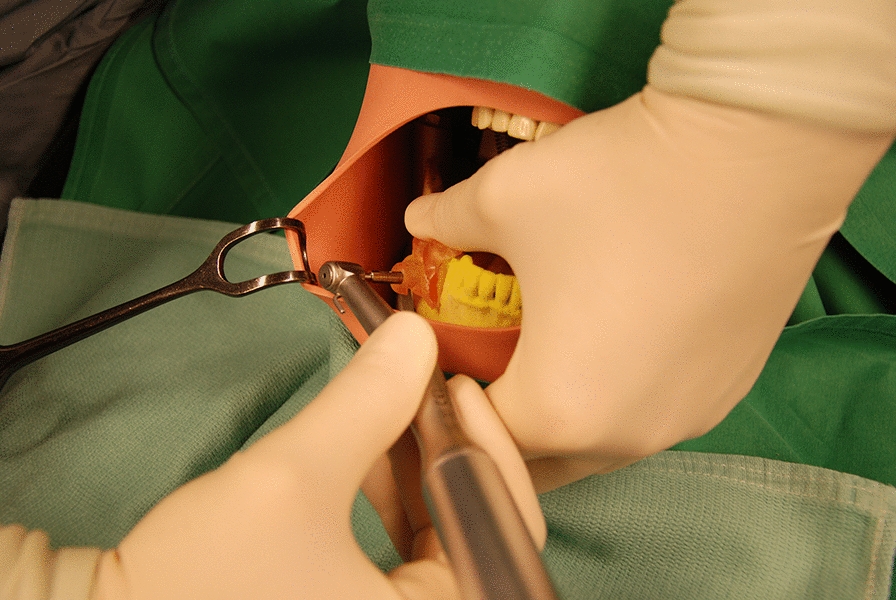
Biopsy in the control group

The models of the lower jaw were fitted into a phantom head model which was placed on the operating table (Frasaco P6 phantom head, frasaco GmbH, Tettnang, Germany) to simulate operating room conditions (Fig. 4). With the help of the surgical template the biopsy was taken (Fig. 5) with a trephine drill (XiVE Trephine Drill, inner diameter 3.0 mm, outer diameter 4.2 mm, Dentsply, York, PA, USA). All guided biopsies were performed by the same experienced surgeon.

### Freehand biopsies/control group

**Fig. 6:**
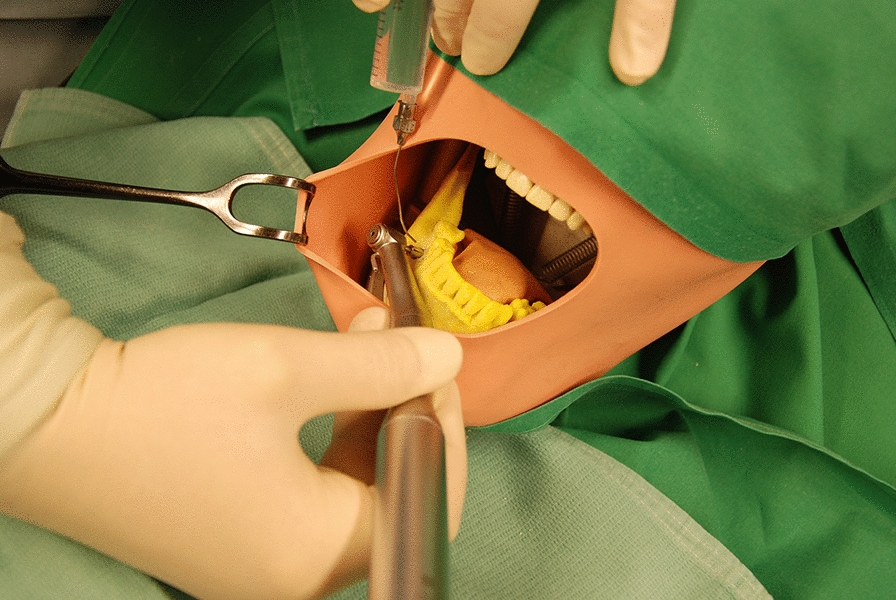
Biopsy in the freehand group

Again, the models of the lower jaw were fitted into a phantom head model (Frasaco P6 phantom head, frasaco GmbH, Tettnang, Germany). Freehand biopsies were taken (Fig. 6) with a trephine drill (XiVE Trephine Drill, inner diameter 3.0 mm, outer diameter 4.2 mm, Dentsply, York, PA, USA). Freehand biopsies were performed about 1 month after the guided biopsies by the same surgeon as for the guided biopsies.

### CT scans after operation/experiments

Again, CT scans the models of the 3D-printed jaws were performed with a Siemens Somatom Force CT scanner (120 kV, 330 mAs, collimation 64 × 0.6 mm, pitch 0.55, slice 0.75 mm).

### Evaluation

**Fig. 7:**
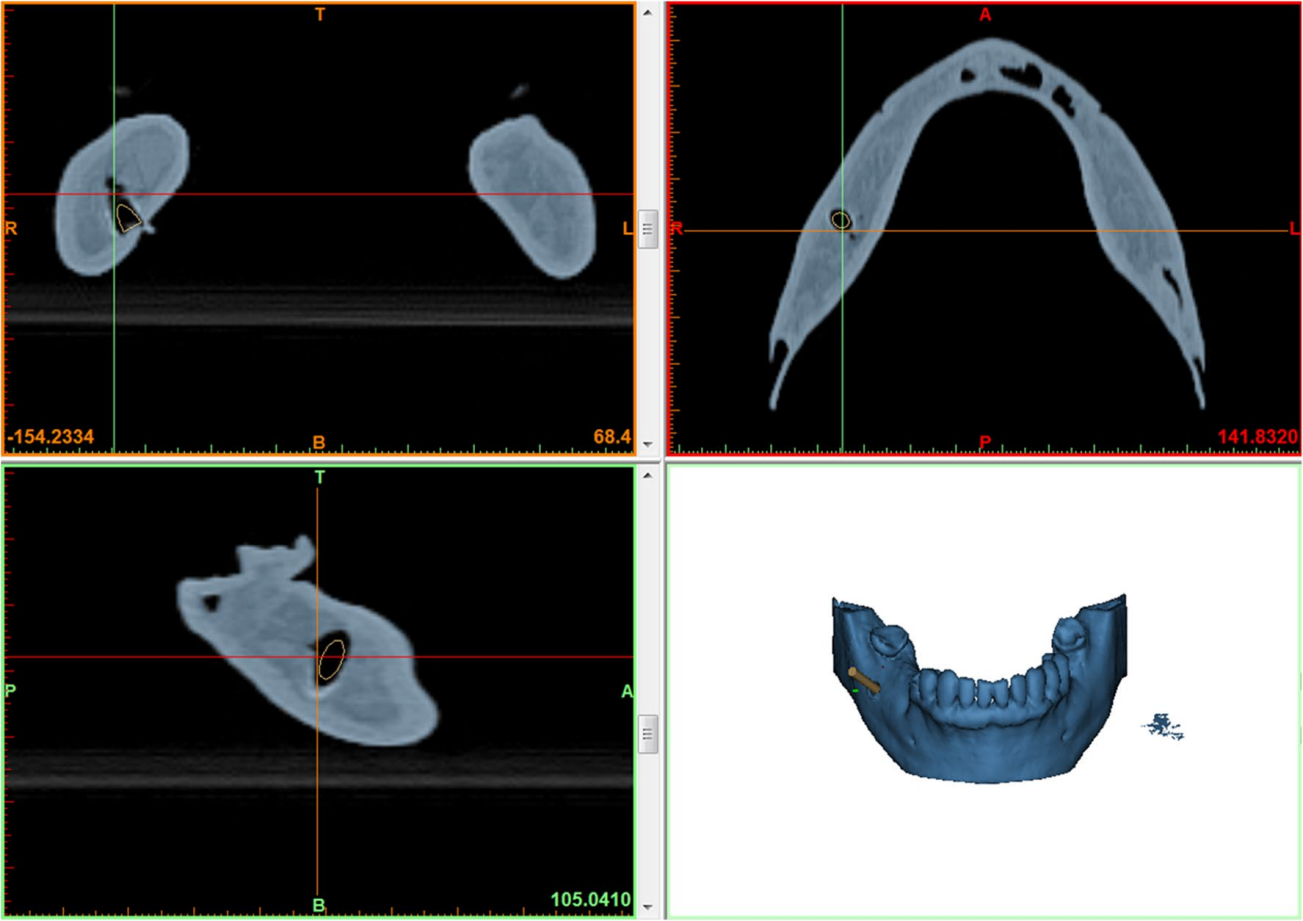
Part of a screenshot while determination of the biopsy channel

**Fig. 8:**
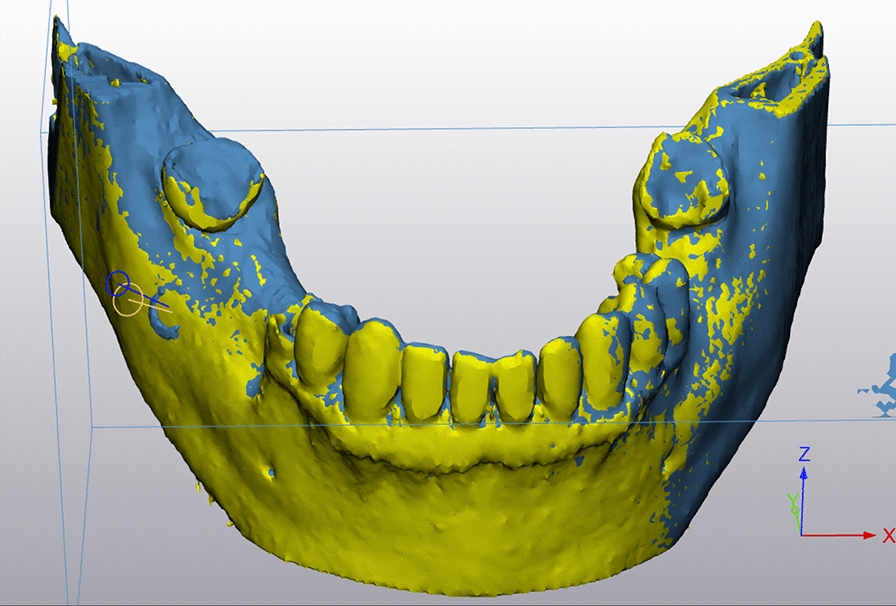
Part of a screenshot after fusion of postoperative data with preoperative planning data for measuring the maximum deviation of axes, of biopsy depth and the angle between the axes

**Fig. 9:**
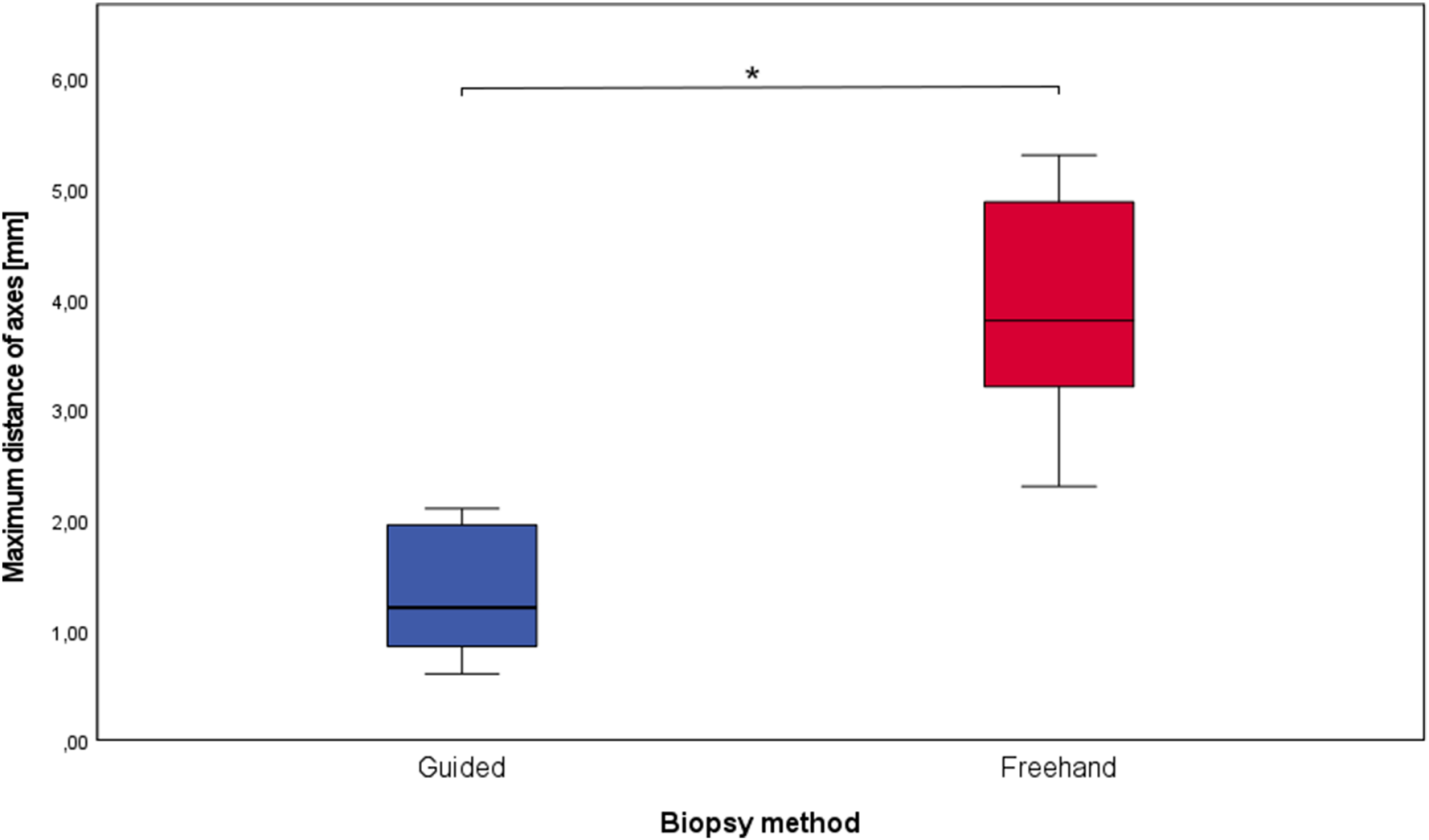
Box plot of the maximum deviation of axes for the guided and the freehand group. The boxes show the range from the first to the third quartile (*p = 0.0002)

The postoperative CT scans’ data were segmented and loaded into Mimics Innovation Suite software (Materialise, Leuven, Belgium). The true biopsy channel was determined (Fig. 7). A fusion of these postoperative data with the preoperative planning data was performed (Fig. 8). The deviation of the planned biopsy channels to channels drilled in reality was determined by measuring the maximum distance in mm between the axes. Measuring the maximum distance in mm is in accordance with the ISO 1101 standard [11] and has been previously used for determining the accuracy of tumor operations [12, 13]. The angle between the actual biopsy axis and the planned biopsy axis was also determined as well as the depth of the biopsy channels which was compared to the planned depth (Fig. 9).

### Statistics

Values are given as mean values (arithmetic mean) and standard deviations which were calculated with the software IBM SPSS Statistics 22 (IBM, Armonk, NY, USA). The proof of normal distribution was performed with the software Sigma Stat 3.1 software (Systat Inc, Chicago, IL, USA). For the comparison of the biopsies using surgical templates and the freehand biopsies, the Welch’s *t* test (two-sample unpooled *t* test for unequal variances) was performed with the QuickCalcs software (GraphPad Software, Inc. La Jolla, CA, USA).

## Results

The mean deviation of the biopsy axes (as described in the materials and methods section) turned out to be 1.3 ± 0.6 mm for the biopsies with a surgical guide and 3.9 ± 1.1 mm for the freehand biopsies (Fig. 9). The data were normally distributed and statistical comparison of the groups revealed a significant difference (*p* value = 0.0002).

**Fig. 10:**
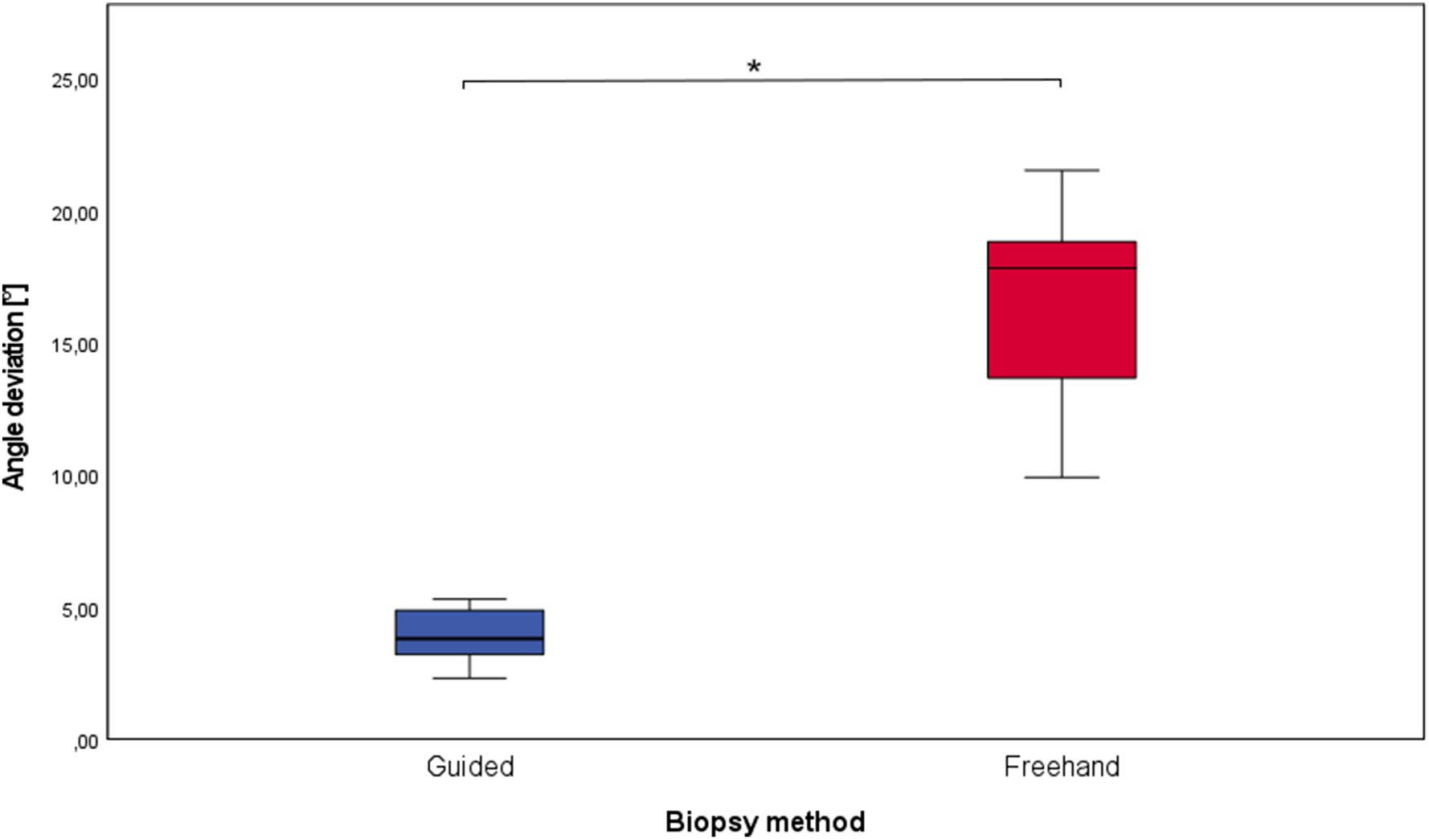
Box plot of the maximum deviation of axes for the guided and the freehand group. The boxes show the range from the first to the third quartile (*p = 0.0013)

The average biopsy angle deviation was 7.7 ± 4.8° for template biopsies and 16.5 ± 3.8° for freehand biopsies (Fig. 10). The difference of the normally distributed data was again statistically significant (*p* value = 0.0013).

**Fig. 11:**
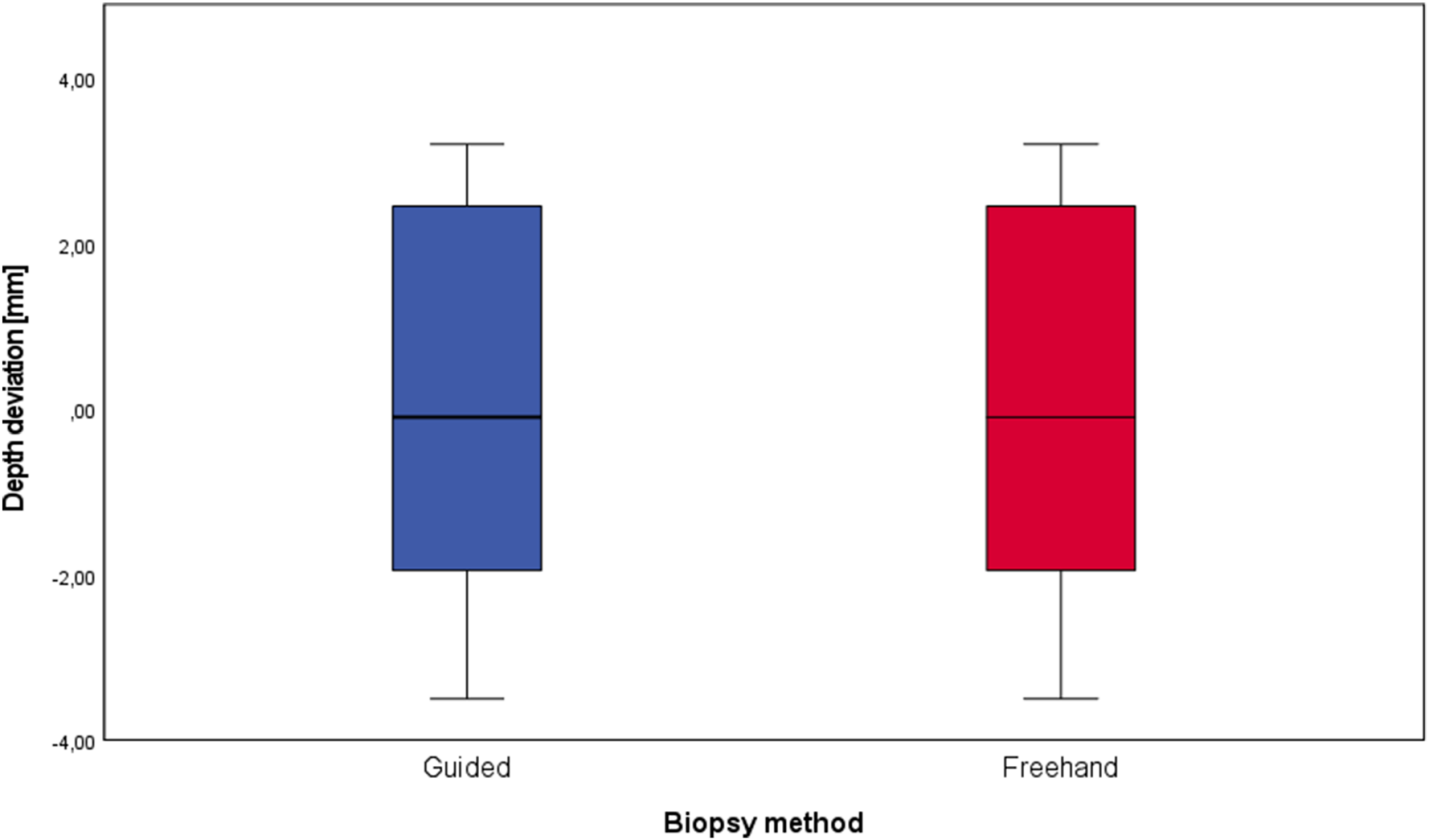
Box plot of the biopsy depth for the guided and the freehand group. The boxes show the range from the first to the third quartile (*p = 0.85)

The difference between the planned depth of biopsy channels and the actual depth of biopsy channels was 0.1 ± 2.5 mm for biopsies with surgical guides and 0.4 ± 3.5 mm for freehand biopsies (Fig. 11). These data showed a normal distribution again, but no statistically significant difference was found (*p* value = 0.85). The time required to fabricate the surgical guides was 128 ± 17 min.

The data are summarized in the following table (Table 1).

**Table 1:** Deviation values for biopsies with surgical guides and freehand biopsies

	Guided biopsies	Freehand biopsies
Maximum distance of axes	1.3 ± 0.6 mm	3.9 ± 1.1
Angle deviation	7.7 ± 4.8°	16.5 ± 3.8°
Depth deviation	0.1 ± 2.5 mm	0.4 ± 3.5 mm

## Discussion

This study evaluated the accuracy of in-house 3D-printed surgical guides for biopsies of the lower jaw. Customized 3D-printed models of the lower jaws were used.

The maximum distance of axes turned out to be 1.3 ± 0.6 mm in the guided group and was significantly lower than in the freehand group (3.9 ± 1.1 mm). The angle between the planning and the actual biopsy was significantly lower in the computer-aided group (7.7 ± 4.8°) compared to the freehand group (16.5 ± 3.8°). A significant difference in the accuracy of biopsy depth could not be found when comparing the study group to the control group. No complications or problems occurred during the biopsy procedure in both the study and control group. Due to the lack of data in the literature concerning biopsies with surgical templates, the results of this study can most likely be compared to studies that have examined the accuracy of implant placing with surgical guides printed by stereolithography. Lee et al. report a mean deviation of implants of < 1 mm and mean angular deviation < 3°, however, they measured the distance at the coronal (top) side of the implants [10]. This method of measuring makes sense for implants, but for biopsies the deviation at the end of the biopsy channel is of greater interest. Cassetta et al. found apical deviations of 2.2 ± 0.8 mm and angulation deviations of 4.7 ± 2.7° in their implant study [14]. Van de Wiele et al. reported apical deviations of 1.60 ± 1.7 mm and a difference in angulation of 2.71 ± 2.7° [6]. These deviations are within the range of the present study’s results. The deviation of angles seems higher in our biopsy study compared to evaluations on implants. We suspect that the reason for the increased deviation could be the usually greater distance between the drill channel and the teeth, since the templates in the studies were tooth-supported. The use of templates did not significantly improve the accuracy of biopsy channel depth. This shows that even visual reading of the scale on the trephine drills can be sufficiently accurate.

The use of surgical templates for implant positioning is progressively becoming more popular [15–17] and the accuracy of these systems has been recently evaluated by several authors [5–7, 9, 10, 14, 18–24]. Among these evaluations of implant positioning, some of the recent studies also used surgical guides that were printed with a stereolithographic 3D printer [6, 9, 10, 14]. Since the medical technology industry provides these promising systems for dental implants, it is questionable whether these systems are also suitable for an off-label use to perform biopsies of jaws. Until recently, stereolithographic printers with a reasonable resolution, an adequate build volume and biocompatible resins were either not available or not affordable. The desktop printer used in this study has an axis resolution of 0.025 mm and a build volume of 125 × 125 × 165 mm and is, therefore, suitable for producing surgical guides. The printer is able to process a class 1 biocompatible resin. This easily allows the production of a surgical guide which can be used in patients. In our case, the templates were produced in-house, which represents a time-saving and resource-efficient procedure. It has to be considered that conventional surgical guides produced without CAD-CAM technology by dental laboratories are common tools and widely used in dentistry for positioning implants [23]. For dental implants, the axis of implantation can often be determined or approximated via the surface information of the teeth, e.g., from a plaster model [23]. However, a biopsy most importantly requires the osseous information with the location of the lesion, which is needed in addition to the surface information. For the fusion of the osseous information with the surface information, CAD environment is an important tool [25]. Planning of the surgical guide usually results in a very complex 3D body that can be best produced via CAM technology. Thus, CAD/CAM is a very helpful technology for biopsies, and therefore, we think that it is important to evaluate the feasibility and accuracy of this technology in the field of oral surgery.

Dense objects can cause metal artifacts, and therefore, extensive metal dental restorations can complicate planning, which is a limitation of the method [26, 27]. Using 3D-printed lower jaws instead of patients surely presents another limitation of this study. Without soft tissue, the dental and bone geometries were clearly visible and accessible. In contrast, the present control group also has advantages. The dental and osseous geometry of the 3D-printed lower jaws was identical to the lower jaws of real patients with lesions that needed biopsies. This method allowed us to use the same planning for the study group and the control group. This way the potential bias due to different locations and directions of the biopsies and different geometries of the jaws was reduced. Since the freehand osteotomies were performed after the guided biopsies, the surgeon may have already gained an advantage performing the freehand biopsies through practice with the guided biopsies. It can, therefore, be assumed that the accuracy of the freehand biopsies would have been even lower if they had been performed in a completely independent control group. This implies that the statistical significance between guided and freehand biopsies is most likely even higher than we evaluated in this study. The errors of the single steps (e.g., scanning, fusion, printing the guides) were not evaluated. However, this study should evaluate the final result.

## Conclusions

From this study, we conclude that surgical guides that were produced with a stereolithographic desktop 3D printer allow significantly higher accuracy of biopsies. Since there were no data in the literature on the accuracy of templates for biopsies in the jaw region, this study is of high relevance. However, the method must now also be investigated in a clinical case series.

## Data Availability

Not applicable.

## Abbreviations

CAD: Computer-aided design
CAM: Computer-aided manufacturing
CT: Computed tomography
DICOM: Digital imaging and communications in medicine (file format)
ISO: International organization for standardization
STL: Standard tessellation language (file format)
